# Is There a Correlation Between Inflammatory Markers and Anti-Müllerian Hormone Level in Non-Obese Patients with Polyendocrine Metabolic Ovarian Syndrome?

**DOI:** 10.3390/biomedicines14102215

**Published:** 2026-09-30

**Authors:** Dominika Gałczyńska, Natalia Przybyła, Kinga Wołkowska, Teresa Wójtowicz, Kamil Kiecka, Dagmara Pluta, Grzegorz Franik

**Affiliations:** 1 Department of Gynecological Endocrinology, Faculty of Medical Sciences in Katowice, Medical University of Silesia, 40-752 Katowice, Poland; 2 Department of Medical and Molecular Biology, Faculty of Medical Sciences in Zabrze, Medical University of Silesia, 40-752 Katowice, Poland

**Keywords:** PMOS, PCOS, inflammation markers, AMH, low-grade inflammation

## Abstract

**Background**: The relationship between low-grade inflammation and anti-Müllerian hormone (AMH) in Polyendocrine Metabolic Ovarian Syndrome (PMOS) remains incompletely understood. This study aimed to compare inflammatory markers and AMH concentrations between non-obese women with PMOS and controls and to assess the associations between these markers and AMH. **Methods**: This single-centered retrospective study involved 92 patients divided into two groups: a control group of 45 patients and 47 patients newly diagnosed with PMOS according to Rotterdam criteria. **Results**: Compared with controls, patients with PMOS had significantly higher median concentrations of CRP (3.902 vs. 1.038), IL-6 (4.15 vs. 2.28), and TNF-α (3.35 vs. 1.95), as well as higher white blood cell and lymphocyte counts. Median AMH (6.47 vs. 3.04), LH (8.77 vs. 6.93), and testosterone (0.415 vs. 0.317) concentrations were also significantly higher in the PMOS group. Conversely, NLR and PLR were higher in controls. When both groups were analyzed together, AMH correlated positively with IL-6 (*r* = 0.53), CRP (*r* = 0.53), TNF-α (*r* = 0.54), LH (*r* = 0.50), and testosterone (*r* = 0.44). In the pooled cohort, AMH correlated positively with CRP, IL-6, and TNF-α. Although TNF-α was significantly associated with AMH in the initial multivariable quantile regression models, these associations were no longer statistically significant after adjustment for PCOS/control status. Group status remained significantly associated with AMH at the 90th percentile. **Conclusions**: No statistically significant correlations were found between AMH levels and inflammatory markers in non-obese patients with PMOS or in healthy women.

## 1. Introduction

Polycystic Ovary Syndrome (PCOS) is a complex and heterogeneous endocrine disorder, commonly occurring among women of reproductive age. In addition to its characteristic reproductive and endocrine manifestations, including hyperandrogenism, ovulatory dysfunction, and polycystic ovarian morphology, PCOS is associated with metabolic, cardiovascular, and immune system disturbances. These diverse manifestations highlight the multisystemic nature of the condition and the complexity of its incompletely understood pathophysiology [1].

Recently, it has been proposed to change the name of Polycystic Ovary Syndrome to Polyendocrine Metabolic Ovarian Syndrome (PMOS), which better captures the nature of this medical condition and the challenges patients face [1]. For the purposes of this article, in light of current guidelines and cited literature, the terms PCOS and PMOS will be used interchangeably, while acknowledging the ongoing changes in relevant nomenclature.

Published studies suggest that the multifactorial nature of the patomechanisms in PMOS manifests itself as an imbalance of cytokines involved in modulating the immune response with a predominance of pro-inflammatory cytokines, such as interleukin 6 (IL-6), tumor necrosis factor alpha (TNF-α) and interleukin 18 (IL-18) [2,3]. Intensification of their synthesis is related to hyperinsulinemia, insulin resistance, and metabolic disorders, and is additionally stimulated by the pro-inflammatory activity of adipose tissue and the presence of elevated levels of advanced glycation end-products (AGE) and increased expression of receptors for those products. Enhanced glycation and mitochondrial dysfunction lead to disruption of the oxidant–antioxidant balance, resulting in a predominance of oxidative activity and the production of reactive oxygen species (ROS) resulting in increased oxidative stress. As a result of these processes, in patients with PMOS, elevated laboratory markers indicative of low-grade inflammation are present, such as CRP or white blood cells (WBC). However, their limited specificity should be taken into account [4]. Additional hematological indices, including mean platelet volume (MPV), red blood cell distribution width (RDW), the neutrophil-to-lymphocyte ratio (NLR), and the platelet-to-lymphocyte ratio (PLR), have also been proposed as indirect indicators of systemic inflammation. These parameters may reflect alterations in inflammatory and hematological responses, although their diagnostic utility in PCOS remains uncertain [5].

Anti-Müllerian hormone (AMH), produced by the granulosa cells of developing ovarian follicles, plays an important role in folliculogenesis and is widely used as a marker of ovarian reserve. Women with PCOS frequently exhibit elevated serum AMH concentrations, primarily associated with an increased number of small antral follicles. Elevated AMH concentrations may contribute to impaired follicular development by reducing follicular sensitivity to follicle-stimulating hormone (FSH) and inhibiting aromatase activity, potentially promoting anovulation and hyperandrogenism [2,3]. However, the interpretation of AMH as a marker of ovarian reserve in women with PCOS remains challenging, as increased concentrations may reflect an excessive number of small follicles rather than their functional capacity.

The available literature regarding the impact of chronic inflammation on ovarian reserve remains limited; therefore, the aim of this single-center, retrospective study was to compare circulating inflammatory markers, including CRP, IL-6, TNF-α, NLR, PLR, MPV, and RDW, as well as AMH concentrations, between non-obese women with PCOS phenotype A (*n* = 47) and healthy controls (*n* = 45). Furthermore, we aimed to investigate the associations between AMH concentrations and inflammatory markers both in the overall study population and separately within each group. By focusing exclusively on women with PCOS phenotype A and excluding overweight and obese participants, we sought to reduce clinical heterogeneity and examine these associations in a more homogeneous study population.

## 2. Materials and Methods

A single-center retrospective study involving 92 patients, divided into two groups: a control with 45 patients and 47 patients newly diagnosed with PCOS/PMOS according to the Rotterdam criteria, was conducted in the Department of Endocrinological Gynecology between October 2025 and June 2026. The sample size was determined by the number of eligible participants recruited during the study period. The inclusion criteria for the control group were: women aged 18–40 years, not using any kind of hormonal contraception for at least 3 months, having regular menstruation (28 +/− 5 days), without any current, acute infection/inflammatory process (especially respiratory, urinary or vaginal) or being more than 2 weeks after an acute infection. The exclusion criteria were: overweight or obesity, taking antibiotics or anti-inflammatory medications chronically, vaccination within 4 weeks, hypothyroidism, taking psychotropic drugs, cigarettes, alcohol or other psychoactive substance abuse, and autoimmune diseases (including systemic connective tissue diseases; thyroid diseases, inflammatory bowel disease, adrenal disorders, skin diseases such as psoriasis and alopecia areata, autoimmune hepatitis, celiac disease, Addison–Briemer anemia, sarcoidosis, and type 1 diabetes). The study group consisted of women aged 18–40 years with a confirmed diagnosis of PCOS based on two out of the three following Rotterdam criteria: anovulatory cycles (irregular cycles < 21 days or >35 days), hyperandrogenism (clinical: presence of acne or hirsutism; or biochemical: above the normal range for testosterone (free or total), dehydroepiandrosterone sulfate (DHEAS), or androstendione) and polycystic ovaries (>20 follicles measuring 2–9 mm in diameter and/or an ovarian volume > 10 mL in at least one ovary). There are four subtypes of PCOS, but for the purpose of this study only patients with subtype A called “classic PCOS”, meeting all three Rotterdam criteria, were included. This selection was intended to reduce clinical heterogeneity and assess markers within a consistently defined phenotype. Excluded from this study were patients diagnosed with PCOS suffering from overweight or obesity, Cushing’s syndrome, hyperprolacinemia, congenital adrenal hyperplasia, hypothalamic–pituitary dysfunction or insufficiency, acromegaly, premature ovarian insufficiency, adrenal tumors and any autoimmune diseases, including Hashimoto’s disease [6,7].

All participants of the study had their venous blood tested between the 2nd and the 6th day of their menstrual cycle. Patients from the control group had their blood drawn once for the following hormones: glucose and insulin after fasting, AMH, DHEAS, FSH, LH, testosterone, cortisol, prolactin (PRL), thyroid-stimulating hormone (TSH) and a complete blood count including WBC, platelet count (PLT), lymphocytes, neutrophils, NLR (neutrophil-to-lymphocyte ratio), PLR (platelet-to-lymphocyte ratio), mean platelet volume (MPV), CRP, Il-6, TNF-a. Study group patients had their blood drawn every day throughout a 4-day hospitalization. On the first day, the following hormones were measured at 10 AM: FSH, LH, AMH, PRL, androstendione, DHEAS, 17-OH-progesterone, free and total testosterone and sex hormone binding globulin (SHBG). On the second day, after 12 h of fasting, glucose, insulin, TSH, complete blood count (CBC), lipid profile and liver enzymes were checked. Subsequently, on the same day, an oral glucose tolerance test (OGTT) was performed, measuring glucose at 0′ and 120′, with cut-off values accordingly 99 mg/dL and 140 mg/dL. Furthermore, on that day, vaginal ultrasound screening took place. On the third day blood was drawn for cortisol at 8.00 AM and 11.00 PM; later on, the patients took 1 mg of dexamethasone. On the last day of hospitalization we checked the cortisol level after dexamethsaone at 8.00 AM. Venous blood samples were sent to the hospital laboratory every day. To measure levels of CRP, Il-6 and TNF-a the serum was collected in the laboratory and stored at −80 °C, and then transported, under appropriate conditions, to a collaborating laboratory. Clinical assessment of acne was performed using the Acne Severity Index, evaluating the type and number of active lesions on the face, chest and back. The assessment accounted for individual acne lesion types, with scores assigned according to lesion severity. Scores ranged from 0 to 5, where 0 indicated the absence of acne lesions and 5 indicated the presence of pustules, infiltrates, and active inflammatory papules in each of the body regions listed above [8]. The Ferriman–Gallwey Score was the tool used to assess hirsutism as a feature of hyperandrogenism. It involves nine areas of the body, where each area is scored from 0 (no hair) to 4 (extensive hair growth) points. A total score of 8 or more points indicates hirsutism in Caucasian women.

AMH concentrations were measured using the Electrochemiluminescence Immunoassay (ECLIA) method on a Cobas Pro Analyzer with the Elecsys AMH Plus reagent (Roche Diagnostics, Mannheim, Germany). Serum CRP concentrations were determined using an enzyme-linked immunosorbent assay (ELISA) with the Human CRP kit (Cat. No. K9710S, Immundiagnostik AG, Bensheim, Germany). The assay was performed according to the manufacturer’s instructions. Absorbance was measured at a wavelength of 450 nm with a reference wavelength of 620 nm using a SYNERGY H1 microplate reader (BioTek, Winooski, VT, USA). Data were analyzed using Gen5 software version 3.05 (BioTek, Winooski, VT, USA). The analytical sensitivity of the assay was 1.015 ng/mL, and the intra-assay precision, expressed as the coefficient of variation (CV), was 6.8%. Serum IL-6 concentrations were determined using an enzyme-linked immunosorbent assay (ELISA) with the Human IL-6 kit (Cat. No. RD194015200R, BioVendor Group, Brno, Czech Republic). The assay was performed according to the manufacturer’s instructions. Absorbance was measured at a wavelength of 450 nm using a SYNERGY H1 microplate reader (BioTek, Winooski, VT, USA). Data were analyzed using Gen5 software version 3.05 (BioTek, Winooski, VT, USA). The analytical sensitivity of the assay was 0.32 pg/mL, and the intra-assay precision, expressed as the coefficient of variation (CV), was 7.1%. Serum TNF-alpha concentrations were determined using an enzyme-linked immunosorbent assay (ELISA) with the Human TNF-Alpha kit (Cat. No. RAF128R, BioVendor Group, Czech Republic). The assay was performed according to the manufacturer’s instructions. Absorbance was measured at a wavelength of 450 nm with a reference wavelength of 620 nm using a SYNERGY H1 microplate reader (BioTek, Winooski, VT, USA). Data were analyzed using Gen5 software version 3.05 (BioTek, Winooski, VT, USA). The analytical sensitivity of the assay was 2.3 pg/mL, and the intra-assay precision, expressed as the coefficient of variation (CV), was 7.2%. Vaginal ultrasound was conducted using Voluson 730 Expert (GE Healthcare, Zipf, Austria).

Statistical analyses were performed using R (R Foundation for Statistical Computing, Vienna, Austria) in the RStudio environment (Posit Software, PBC, Boston, MA, USA). The normality of continuous variables was assessed using the Shapiro–Wilk test (Table 1). Between-group comparisons were performed using Student’s *t*-test for approximately normally distributed variables and the Wilcoxon rank-sum test for the remaining variables. Comparisons performed using Student’s *t*-test are marked with an asterisk in Table 2. Correlations were assessed using Spearman’s rank correlation coefficient (ρ), both in the pooled sample and separately within the PCOS and control groups. *p*-values < 0.05 were considered statistically significant.

Univariable median quantile regression models were fitted to assess differences in the conditional median of each analyzed parameter between the PCOS/PMOS and control groups (Table 3). Each parameter was entered as the dependent variable in a separate model, with group status as the sole predictor and the control group as the reference category. To investigate associations between individual inflammatory and hematological parameters and AMH concentrations, separate univariable quantile regression models were fitted at the 10th, 50th, and 90th conditional percentiles of AMH in the pooled sample (Table 4). Each model included AMH concentration as the dependent variable and a single parameter as the independent variable. Exploratory multivariable quantile regression models were fitted at the 10th, 50th, and 90th conditional percentiles of AMH to investigate associations between AMH concentrations and inflammatory and hematological parameters. The initial multivariable models included TNF-α, NLR, PLR, and MPV as simultaneous independent variables. To assess whether the observed associations persisted after accounting for differences between women with PCOS and healthy controls, additional multivariable quantile regression models were fitted using the same predictors, with PCOS/control status included as an additional covariate. The PCOS group was used as the reference category. The group-adjusted models are presented in Table 5, while the corresponding models without adjustment for PCOS/control status are provided in Supplementary Table S1. Additional exploratory multivariable quantile regression analyses were performed separately in the PCOS and control groups using the same four predictors (TNF-α, NLR, PLR, and MPV). The results of these stratified analyses are presented in Supplementary Table S2. Regression results are presented as regression coefficients, with corresponding standard errors, 95% confidence intervals, and *p*-values.

All methods were performed in accordance with the guidelines and regulations of the Department of Gynecological Endocrinology, Medical University of Silesia. Informed consent was obtained from all study participants.

## 3. Results

Table 1 presents characteristics for the entire study population, including both patients with PCOS/PMOS and controls, without stratification by group. In the study group of patients with PCOS/PMOS, there was a statistically significantly increased level of inflammatory markers, i.e., CRP (median 3.902 vs. 1.038), Il-6 (4.15 vs. 2.28), TNF-alpha (3.35 vs. 1.95), WBC (6.16 vs. 5.2), limfocytes (2.57 vs. 1.88) and hormones including AMH (6.47 vs. 3.04), LH (8.77 vs. 6.93), testosterone (0.415 vs. 0.317), and decreased serum levels of glucose (85.2 vs. 88.8), insulin (5.68 vs. 6.97), FSH (5.76 vs. 6.5), PRL (9.49 vs. 16.7) and BMI (21 vs. 21.94), whereas in the control group NLR (131.07 vs. 93.17) and PLR (137.99 vs. 101.08) were higher than in the study group. No statistically significant difference was determined between the groups with respect to other markers of inflammation: PLT, neutorphils, MPV and RDW (Table 2). All 47 women in the PCOS group met both clinical (modified Ferriman–Gallwey score ≥ 8) and biochemical criteria for hyperandrogenism. None of the 45 women in the control group met either criterion.

**Figure 1:**
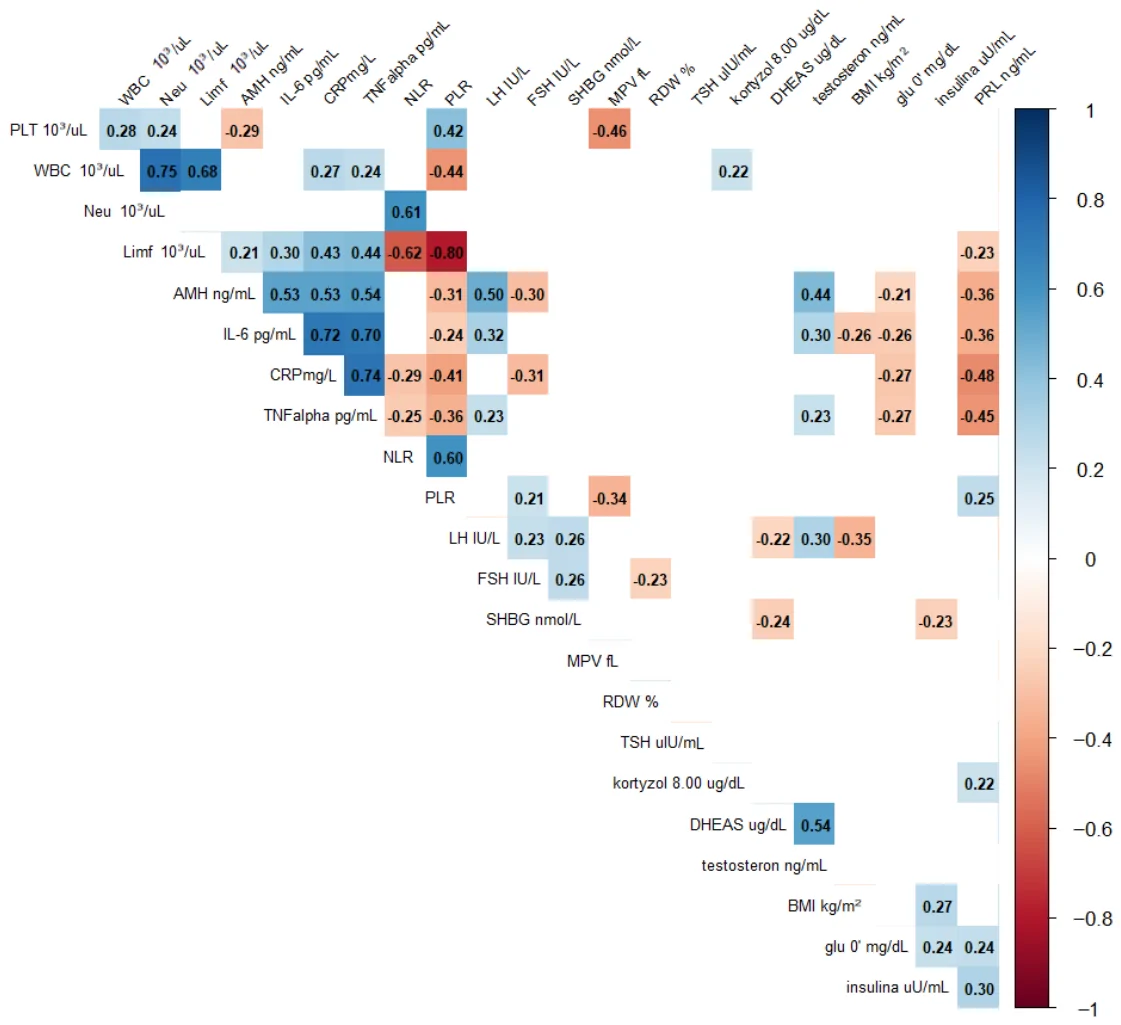
Heatmap plot presenting correlations in the overall cohort. Abbreviations: CRP—C-reactive protein, Il-6—Interleukin 6, TNF-alpha—tumor necrosis factor alpha, AMH—anti-Müllerian, DHEA-S—dehydroepiandrosterone sulfate, FSH—follicle-stimulating hormone, LH—luteinizing hormone, T—testosterone, Cort—cortisol, SHBG—sex-hormone binding globulin, PRL—prolactin, TSH—thyroid-stimulating hormone; WBC—white blood cell, PLT—platelet count, Limf—limphocyte, Neu—Neutrophils, NLR—neutrophils/lymphocytes ratio, PLR—platelet/lymphocyte ratio; MPV—mean platelet volume, RDW—red blood cell distribution width; BMI—body mass index.

**Figure 2:**
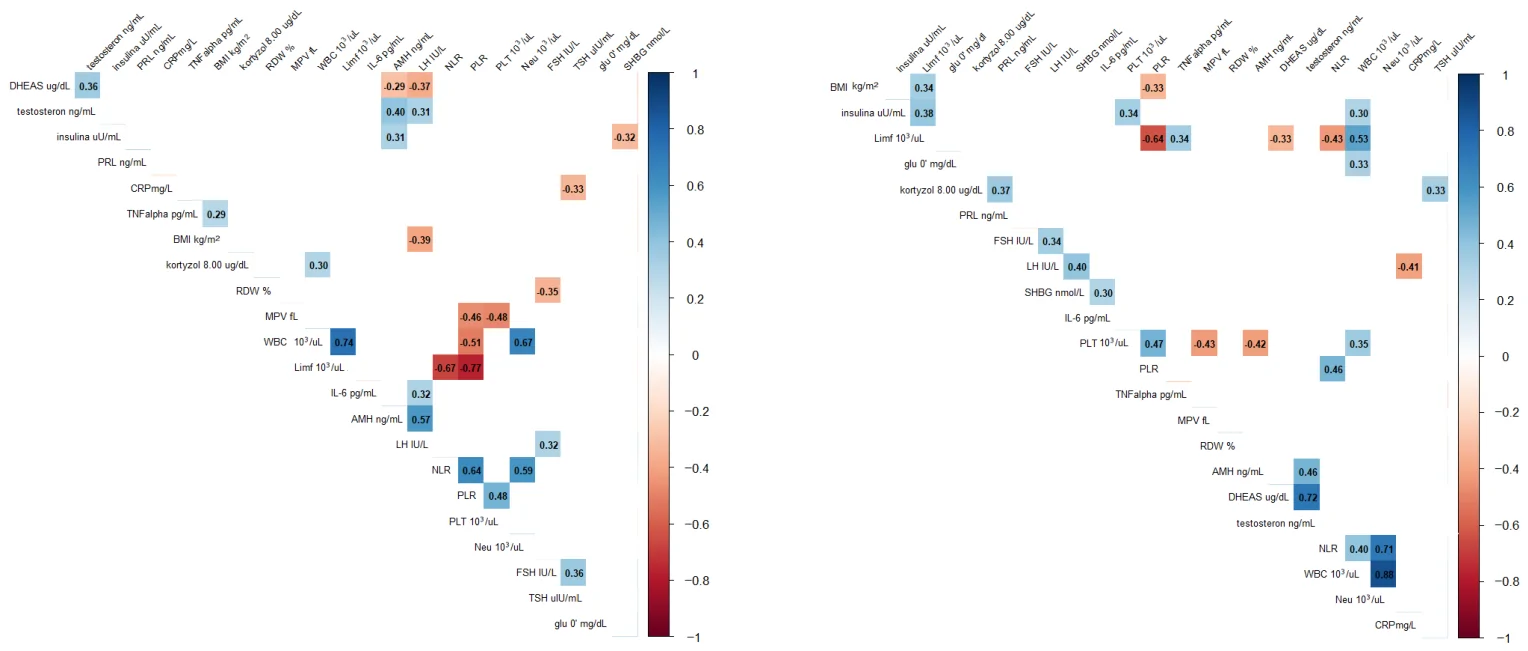
Heatmap plot presenting correlations in the study group (**left** side) and in the control group (**right** side). Abbreviations: CRP—C-reactive protein, Il-6—Interleukin 6, TNF-alpha—tumor necrosis factor alpha, AMH—anti-Müllerian, DHEA-S—dehydroepiandrosterone sulfate, FSH—follicle-stimulating hormone, LH—luteinizing hormone, T—testosterone, Cort—cortisol, SHBG—sex-hormone binding globulin, PRL—prolactin, TSH—thyroid-stimulating hormone; WBC—white blood cell, PLT—platelet count, Limf—lymphocyte, Neu—Neutrophils, NLR—neutrophil/lymphocyte ratio, PLR—platelet/lymphocyte ratio; MPV—mean platelet volume, RDW—red blood cell distribution width; BMI—body mass index.

Correlations between inflammatory markers and hormones in the overall study population, without stratification by group, are presented as follows in Figure 1. A positive correlation of AMH with Il-6 (*ρ* = 0.53), CRP (*ρ* = 0.53), TNF-a (*ρ* = 0.54), LH (*r* = 0.50), and testosterone (*ρ* = 0.44) was observed. In addition, negative correlations were found with FSH (*ρ* = −0.30), PLR (*r* = −0.31), glucose (*ρ* = −0.21) and PRL (*ρ* = −0.36). When patients were divided into the control and study groups, there were no correlations between AMH and inflammatory markers (Figure 2).

The results of the univariate quantile regression analysis at the 50th percentile indicate a statistically significant association between the parameters presented below. Positive associations were observed for CRP, IL-6, TNF-α, and AMH concentrations. The PMOS group had a median AMH concentration 3.43 ng/mL higher than the control group (95% CI: 1.359–5.501; *p* = 0.001). At the same time, statistically significant negative associations were observed for PRL and the PLR. For PRL, the estimate was −7.21 (95% CI: −11.404 to −3.016; *p* = 0.001), whereas for the PLR, the estimate was −37.903 (95% CI: −64.575 to −11.230; *p* = 0.006) (Table 3).

The results of the univariate quantile regression models at the 10th, 50th, and 90th percentiles (Table 4) were used to assess the association of the analyzed variables with AMH concentration in all patients. CRP concentration was positively associated with AMH across all analyzed percentiles: the 10th percentile (estimate = 0.77; *p* = 0.001), the 50th percentile (estimate = 1.16; *p* = 0.001), and the 90th percentile (estimate = 2.858; *p* < 0.001). A similar pattern was observed for IL-6 and TNF-α. Among the hematological parameters, statistically significant associations with AMH concentration were observed predominantly at the 90th percentile. A positive association was found for lymphocyte count (estimate = 2.848; *p* < 0.001), whereas negative associations were observed for MPV (estimate = −2.60; *p* = 0.020), NLR (estimate = −2.307; *p* = 0.038), neutrophil count (estimate = −2.754; *p* = 0.024), PLR (estimate = −0.037; *p* = 0.003), and RDW (estimate = −1.304; *p* = 0.011). No statistically significant association was found between PLT and AMH concentration.

In the initial multivariable quantile regression models without adjustment for PCOS/control status, TNF-α was positively associated with AMH across all analyzed percentiles (Supplementary Table S1). However, after including PCOS/control status as an additional covariate, the association between TNF-α and AMH was no longer statistically significant at any of the analyzed percentiles (Table 5). At the 10th percentile, the estimated coefficient for TNF-α was 0.220 (95% CI: −1.546 to 1.985; *p* = 0.805). Similarly, no statistically significant association was observed at the 50th percentile (estimate = 0.166; 95% CI: −2.086 to 2.419; *p* = 0.884) or the 90th percentile (estimate = −0.140; 95% CI: −4.047 to 3.767; *p* = 0.944). PCOS/control status was significantly associated with AMH at the 90th percentile. Compared with the PCOS group, the control group exhibited lower conditional AMH concentrations (estimate = −9.927; 95% CI: −17.278 to −2.576; *p* = 0.009). No statistically significant association with group status was observed at the 10th percentile (*p* = 0.155) or the 50th percentile (*p* = 0.056). NLR, PLR, and MPV were not significantly associated with AMH in any of the group-adjusted models. Additional multivariable quantile regression analyses performed separately in the PCOS and control groups did not demonstrate a statistically significant association between TNF-α and AMH at any of the analyzed percentiles (Supplementary Table S2). In the PCOS group, MPV showed a negative association with AMH at the 90th percentile (estimate = −5.188; 95% CI: −10.102 to −0.274; *p* = 0.039). No other statistically significant associations were identified in the stratified multivariable models.

## 4. Discussion

In the present study, non-obese women with PCOS phenotype A exhibited significantly higher circulating concentrations of CRP (median 3.902 vs. 1.038 mg/L), IL-6 (4.15 vs. 2.28 pg/mL), and TNF-α (3.35 vs. 1.95 pg/mL) compared with healthy controls. These findings suggest that chronic low-grade inflammatory activation may be present in women with PCOS even in the absence of overweight or obesity. However, the association between TNF-α and AMH observed in the pooled cohort was no longer statistically significant after adjustment for PCOS/control status, indicating that between-group differences may contribute to this association. Our finding of elevated CRP concentrations is consistent with previous studies reporting increased inflammatory activity in women with PCOS [9,10,11,12]. In particular, the systematic review and meta-analysis by Escobar-Morreale et al., which included 31 studies, demonstrated significantly higher circulating CRP concentrations in women with PCOS compared with healthy controls, with this finding persisting after the exclusion of studies with differences in body mass or obesity prevalence between groups [12]. In contrast, the same meta-analysis did not identify statistically significant differences in circulating IL-6 or TNF-α concentrations between women with PCOS and controls, whereas both cytokines were significantly elevated in our study population [12]. These discrepancies may partly reflect differences in study populations, PCOS phenotypes, metabolic characteristics, sample sizes, and laboratory methods. The elevated concentrations of inflammatory markers observed in our cohort may reflect interactions between inflammatory, endocrine, and metabolic pathways involved in PCOS pathophysiology [4]. Nevertheless, the retrospective nature of our study does not allow us to establish causal relationships or determine the relative contributions of individual cytokines to ovarian dysfunction. Further studies are needed to clarify the biological significance of these inflammatory changes in non-obese women with PCOS phenotype A.

Our analysis showed a statistically significant increased level of WBC and lymphocytes in the PMOS compared with the control group (respectively 6.16 vs. 5.2; 2.57 vs. 1.88). In the studies by Orio et al. and Papalou et al., higher WBC counts among PCOS woman were found to be correlated with obesity and insulin resistance, rather than hiperandrogenemia [13,14]. However, other studies detected a positive correlation with androgens, especially testosterone, which, when subjected to multiple regression analysis, was found to be the main predictor factor of leukocyte count [15]. The higher lymphocyte counts observed in our study may indicate a distinct pattern of immune activation in this carefully selected group of non-obese women with PCOS phenotype A. However, the nature of the adaptive immune response cannot be determined from the total lymphocyte count alone. Individual T- and B-lymphocyte subsets may exert different, or even opposing, proinflammatory and anti-inflammatory functions. Due to divergent data on this topic and limited data about lymphocytes as inflammatory markers in the pathogenesis of PMOS, this topic needs further investigation. Perhaps immunophenotyping analyses would be a proper direction to elucidate the biological significance of this finding.

According to our research, NLR and PLR were higher in the control group than in the PMOS group (respectively 131.07 vs. 93.17; 137.99 vs. 101.08). This is a very interesting finding, considering that a meta-analysis by Li Li et al. includes conclusions from 13 studies that NLR is an increased obesity-independent biomarker in PCOS compared to healthy woman [16]. Furthermore, a study by Perglialiotis et al. suggests PLR as a useful tool in the evaluation of low-grade inflammation in PCOS patients [17]. Both parameters in our control group showed increased levels, which contradicts reports in the literature. However, this discrepancy can largely be explained by the structure of the ratio itself. NLR is calculated by dividing the neutrophil count by the lymphocyte count. In our study, neutrophil counts did not differ between the groups, whereas lymphocyte counts were markedly higher in the PCOS group. An increase in the denominator with a stable numerator therefore mathematically results in a lower NLR. Similarly, PLR is calculated by dividing the platelet count by the lymphocyte count. Platelet counts did not differ significantly between the groups, whereas lymphocyte counts were higher in the PCOS group, resulting in a lower PLR. In this context, lower NLR and PLR values should not be interpreted as evidence of less pronounced inflammation. Indeed, CRP, IL-6, TNF-α, and white blood cell counts were higher in the same group. Rather, this finding illustrates the limitations of using composite hematological indices without examining their individual components. NLR and PLR may exhibit different patterns depending on whether the immune response is dominated by neutrophilia, lymphocytosis, changes in platelet counts, or a combination of these processes. This may also explain some of the inconsistencies in the literature. NLR and PLR are nonspecific and susceptible to numerous biological and preanalytical factors. Their values are influenced by infections, stress, physical activity, medications, smoking, the timing of blood sampling, and individual hematological variability. Moreover, no standardized PCOS-specific cutoff values have been established. The findings of the present study do not challenge the presence of chronic inflammation but indicate that NLR and PLR should not be regarded as substitutes for CRP or cytokine measurements.

Patients with PCOS have been shown to exhibit a positive association between serum AMH and LH concentrations, as reported by Dewailly et al., Rudnicka E et al., Pratama G [15,18,19]. This relationship suggests an interaction among AMH, LH, and circulating androgen levels. One possible underlying mechanism of PCOS may involve dysfunction of the hypothalamic–pituitary–ovarian axis, in which altered gonadotropin-releasing hormone (GnRH) pulsatility leads to increased LH secretion from the anterior pituitary. At the same time, reduced FSH levels may alter the LH/FSH ratio and promote increased androgen production by theca cells, ultimately contributing to hyperandrogenism. These hormonal disturbances may also impair normal follicular development, resulting in the accumulation of antral follicles, the development of polycystic ovarian morphology (PCOM), and elevated AMH concentrations [19]. In our study, the results showed a significantly increased level of AMH, LH, and androgens, specifically testosterone (respectively 6.47 vs. 3.04; 8.77 vs. 6.93; 0.415 vs. 0.317), which emphasizes the role of hyperandrogenism in PMOS patients. Hyperandrogenism might, individually or combined with obesity and IR, play an important part in the activation of leukocytes and low-grade inflammation in PMOS. As presented in the literature, chronic low-grade inflammation and IR are closely interrelated. Proinflammatory cytokines can disrupt intracellular insulin signaling. For example, TNF-α may impair normal insulin receptor activation, thereby attenuating the biological effects of insulin. Consequently, glucose uptake by tissues is reduced, and IR is exacerbated. In turn, IR leads to compensatory hyperinsulinemia, which is itself closely associated with metabolic disturbances and inflammation. These processes therefore form a positive feedback loop in which chronic inflammation exacerbates IR, while hyperinsulinemia and the resulting metabolic disturbances promote further activation of proinflammatory pathways [3,4]. Inflammation-associated impairments in insulin signaling have also been reported in women with PCOS of normal body weight, further suggesting that obesity is not the sole source of inflammation in this syndrome. IR and hyperinsulinemia are also associated with hyperandrogenism. Elevated insulin concentrations stimulate ovarian androgen synthesis and reduce hepatic production of sex hormone-binding globulin (SHBG), increasing androgen bioavailability. Conversely, hyperandrogenism may influence adipose tissue distribution, promoting visceral fat accumulation and further exacerbating metabolic disturbances. There is also evidence suggesting a direct association between androgens and leukocyte activity. Androgen receptors are expressed in immune cells, including neutrophils; therefore, hyperandrogenemia may be one of the factors modulating chronic inflammatory activation. These interactions establish a multidirectional relationship among hyperandrogenism, insulin resistance, visceral obesity, and inflammation, in which the individual disturbances may exacerbate one another [3].

In the univariate quantile regression analysis of our results, statistically significant positive associations were demonstrated between AMH and the inflammatory markers CRP, IL-6, and TNF-α. Among the analyzed variables, AMH showed the strongest positive association at the median (50th percentile), with an estimate of 3.43 (95% CI: 1.359–5.501; *p* = 0.001), while PRL and PLR were negatively associated with the outcome. Across the 10th, 50th, and 90th percentiles, CRP was consistently and significantly positively associated with AMH, with the strength of the association increasing at higher AMH concentrations (estimates: 0.77, 1.16, and 2.858, respectively; all *p* ≤ 0.001). A similar positive pattern was observed for IL-6 and TNF-α, suggesting that higher concentrations of these inflammatory markers were associated with higher AMH levels in the cohort. Nevertheless, when the cohort was divided into the study and control groups, no significant correlations were established. This is one of the most important findings of the analysis, as it indicates that the association observed in the overall cohort cannot automatically be interpreted as a continuous biological relationship within the PCOS group itself. There are at least two possible explanations. First, the observed correlation may largely reflect group membership. PCOS is a common factor associated with both higher AMH concentrations and a more proinflammatory profile, without these variables necessarily being directly related to one another. Alternatively, a genuine association may exist but may not have been detected within the individual groups because of their small sample sizes and restricted ranges of values. Dividing the cohort of 92 participants into groups of 47 and 45 reduces statistical power. Furthermore, restricting BMI and selecting a single phenotype reduce biological variability, which may attenuate correlation coefficients. Thus, the stratified analysis cannot be considered to definitively rule out an association between AMH and inflammation. However, it indicates that the evidence for such an association is considerably weaker than the analysis of the overall cohort alone might suggest.

The literature on TNF-α is particularly interesting. An earlier meta-analysis published in 2011 did not confirm a significant increase in this cytokine in PCOS [12], whereas the 2016 meta-analysis by Gao et al., comprising 29 studies and 1960 participants, found significantly higher TNF-α concentrations in women with PCOS. The largest differences were observed in populations characterized by greater insulin resistance and higher testosterone concentrations [20]. A more recent meta-analysis also confirmed a moderate increase in TNF-α, although between-study heterogeneity was very high [21]. TNF-α may be of particular biological relevance because of its role at the interface between the immune system and metabolism. Through its receptors, TNFR1 and TNFR2, TNF-α activates signaling pathways including NF-κB and JNK and may impair normal insulin signaling. Increased activity of serine kinase pathways promotes abnormal phosphorylation of components of the insulin signaling cascade and the development of insulin resistance. Meanwhile, hyperinsulinemia enhances ovarian androgen steroidogenesis, providing a potential mechanism linking inflammation, metabolism, and hyperandrogenism [4]. In the initial multivariable quantile regression models, TNF-α was positively associated with AMH across all three analyzed percentiles. However, these models did not account for PCOS/control status, which may represent an important source of between-group differences. Following additional adjustment for group status, the associations between TNF-α and AMH were substantially attenuated and were no longer statistically significant at any of the analyzed percentiles. These findings suggest that the positive association observed in the original pooled analyses may be largely influenced by differences between women with PCOS and healthy controls rather than reflecting an independent association between TNF-α and AMH. This interpretation is further supported by the additional stratified multivariable quantile regression analyses, in which no statistically significant association between TNF-α and AMH was identified in either group.

Nevertheless, the absence of statistically significant associations in the adjusted and stratified models does not definitively exclude a biological relationship between TNF-α and AMH. The relatively small sample sizes, particularly in the stratified analyses, and the wide confidence intervals limit the precision of the estimates. Interestingly, PCOS/control status remained significantly associated with AMH at the 90th percentile in the group-adjusted model. This finding indicates that between-group differences in the upper part of the conditional AMH distribution persist after accounting for the inflammatory and hematological parameters included in the model. In addition, MPV showed a negative association with AMH at the 90th percentile in the PCOS group. However, given the exploratory nature of the analyses and the multiple statistical comparisons performed, this isolated finding should be interpreted cautiously and requires confirmation in larger studies.

The strengths of this study include highly selected inclusion criteria, homogenous study and control groups, and a highly standardized setup of clinical and laboratory assessments, which minimizes confounding factors. The limitations of this research include a small number of patients. An additional limitation of the present study is the inability to fully reconstruct the original predictor-selection procedure used in the exploratory multivariable regression models. The initial candidate predictor set and the exact criteria used for variable removal could not be verified, limiting the reproducibility of the original variable-selection process. Furthermore, the relatively small sample size and the number of statistical comparisons performed warrant caution when interpreting the findings, particularly the isolated significant association identified in the stratified analyses. The group-adjusted models accounted for PCOS/control status, TNF-α, NLR, PLR, and MPV, but did not simultaneously adjust for all potentially relevant inflammatory, endocrine, and metabolic factors. Therefore, residual confounding cannot be excluded, and the results should be interpreted as exploratory associations rather than evidence of causality.

Therefore, we will seek to address these limitations as much as possible in future research on this and related topics. Future directions of the study might include low-grade chronic inflammation in PMOS patients considered from the perspective of cardiovascular disease risk and potential treatment strategies to minimize morbidity and mortality in this group of patients.

## 5. Conclusions

In non-obese women with PCOS phenotype A, CRP, IL-6, and TNF-α concentrations were significantly higher than in healthy controls, suggesting that chronic low-grade inflammation may be present even in the absence of overweight or obesity. In the pooled cohort, AMH concentrations were positively correlated with CRP, IL-6, and TNF-α; however, these associations were not confirmed when the PCOS and control groups were analyzed separately. Furthermore, the association between TNF-α and AMH was no longer statistically significant after adjustment for PCOS/control status. These findings suggest that between-group differences may substantially contribute to the associations observed in the pooled cohort. Therefore, an independent association between AMH and inflammatory markers in women with PCOS phenotype A could not be confirmed. Further studies with larger sample sizes are needed to clarify the relationship between chronic low-grade inflammation and AMH in women with PCOS.

## Figures and Tables

**Table 1:** Statistical characteristics in the overall cohort.

Variables	N	Min.	Max.	Median	Q1	Q3	Mean	+/−SD
CRP mg/L	92	0.916	5.251	2.775	1.042	3.904	2.517	1.477
IL-6 pg/mL	92	1.3	6.35	3.515	2.302	4.188	3.293	1.13
TNF alpha pg/mL	92	1.47	4.86	2.59	1.95	3.357	2.718	0.796
Glucose 0′ mg/dL	92	74	106	86.2	82.9	90	86.69	5.56
Insulin uU/mL	92	1.99	35.5	6.685	4.592	9.255	7.609	4.738
AMH ng/mL	92	1.12	29.8	4.55	3.055	6.92	5.818	4.551
DHEAS ug/dL	92	33.5	613	260.5	197.75	337.75	279.764	116.786
FSH IU/L	92	1.74	13.2	6.12	5.417	6.9	6.325	1.542
LH IU/L	92	1.73	23.3	7.885	5.777	9.955	8.48	3.928
T ng/mL	92	0.103	0.854	0.357	0.278	0.519	0.386	0.156
SHBG nmol/L	92	16.8	142	61.9	46.025	74.7	62.668	23.077
Cort 8.00 ug/dL	92	7.61	23.2	13.8	11.225	16.5	13.938	3.566
PRL ng/mL	92	4.31	35	13.45	8.47	17.35	14.157	6.51
TSH uIU/mL	92	0.489	4.48	1.96	1.45	2.685	2.112	0.896
WBC 10^3^uL	92	3.09	9.62	5.8	4.853	6.545	5.786	1.313
PLT 10^3^/uL	92	123	381	233.5	209	279	243.565	52.191
Limf 10^3^/uL	92	0.62	4.73	2.095	1.752	2.683	2.247	0.723
Neu 10^3^/uL	92	1.1	5.49	2.7	2.188	3.332	2.789	0.84
NLR	92	0.414	3.323	1.281	0.978	1.568	1.358	0.587
PLR	92	40.592	304.839	111.163	91.118	145.351	119.138	44.07
MPV fL	92	8.7	13.3	10.55	10	11.2	10.652	0.929
RDW %	92	11.3	18.5	12.5	12.2	13.2	12.796	1.05
BMI kg/m^2^	92	17.31	24.9	21.535	19.96	22.762	21.306	1.968

Abbreviations: SD—standard deviation, CRP—C-reactive protein, Il-6—Interleukin 6, TNF alpha—tumor necrosis factor alpha, AMH—anti-Müllerian, DHEA-S—dehydroepiandrosterone sulfate, FSH—follicle-stimulating hormone, LH—luteinizing hormone, T—testosterone, Cort—cortisol, SHBG—sex-hormone binding globulin, PRL—prolactin, TSH—thyroid-stimulating hormone; WBC—white blood cell, PLT—platelet count, Limf—limphocyte, Neu-Neutrophils, NLR—neutrophils/lymphocytes ratio, PLR—platelet/lymphocyte ratio; MPV—mean platelet volume, RDW—red blood cell distribution width; BMI—body mass index.

**Table 2:** Comparison of variables in the control and the study group.

Variables	Group	N	Min	Max	Median	Q1	Q3	Mean	+/−SD	*p*
CRP mg/L	study	47	2.76	5.251	3.902	3.595	4.139	3.894	0.579	<0.001
	control	45	0.916	1.482	1.038	0.997	1.147	1.079	0.137	
IL-6 pg/mL	study	47	2.87	6.35	4.15	3.84	4.485	4.209	0.61	<0.001
	control	45	1.3	4.25	2.28	1.89	2.63	2.336	0.645	
TNF alpha pg/mL	study	47	2.48	4.86	3.35	2.89	3.7	3.36	0.545	<0.001
	control	45	1.47	3.33	1.95	1.87	2.15	2.048	0.317	
Glucose 0′ mg/dL *	study	47	74	96	85.2	82.3	88.7	85.047	5.051	0.003
	control	45	78.1	106	88.8	84.9	91.4	88.407	5.6	
Insulin uU/mL	study	47	1.99	35.5	5.68	4.165	8.56	7.15	5.503	0.038
	control	45	3.31	22.1	6.97	5.97	10	8.088	3.783	
AMH ng/mL	study	47	3.06	29.8	6.47	4.345	9.06	8.058	5.205	<0.001
	control	45	1.12	9.43	3.04	1.89	4.76	3.479	1.884	
DHEA-S ug/dL	study	47	33.5	554	273	194.5	344.5	280.581	125.177	0.824
	control	45	115	613	257	208	330	278.911	108.742	
FSH IU/L	study	47	3.7	10.1	5.76	5.29	6.61	6.039	1.263	0.031
	control	45	1.74	13.2	6.5	5.59	7.41	6.623	1.753	
LH IU/L	study	47	3.14	23.3	8.77	6.405	11.75	9.687	4.644	0.013
	control	45	1.73	12.6	6.93	5.39	8.7	7.22	2.489	
T ng/mL	study	47	0.129	0.854	0.415	0.3	0.556	0.423	0.161	0.015
	control	45	0.103	0.804	0.317	0.257	0.409	0.349	0.142	
SHBG nmol/L	study	47	16.8	119	58.2	46.55	72.25	61.132	22.766	0.519
	control	45	32.3	142	64.5	43.1	78.8	64.273	23.546	
Cort 8.00 ug/dL *	study	47	7.81	23.2	13.8	11.05	16.5	13.96	3.746	0.952
	control	45	7.61	20.3	13.8	11.5	16.3	13.916	3.41	
PRL ng/mL	study	47	4.31	24.5	9.49	7.425	13.7	10.963	5.104	<0.001
	control	45	7.64	35	16.7	13.3	22.7	17.493	6.176	
TSH uIU/mL	study	47	0.489	4.48	2	1.395	2.69	2.109	0.894	0.935
	control	45	0.844	4.2	1.92	1.53	2.68	2.114	0.909	
WBC 10^3^/uL *	study	47	3.58	9.62	6.16	5.505	6.91	6.136	1.282	0.008
	control	45	3.09	8.64	5.2	4.6	6.19	5.42	1.257	
PLT 10^3^/uL *	study	47	136	367	229	209	279	240.213	52.082	0.532
	control	45	123	381	236	213	275	247.067	52.66	
Limf 10^3^/uL *	study	47	1.07	4.73	2.57	2.045	3.145	2.59	0.762	<0.001
	control	45	0.62	3.07	1.88	1.57	2.11	1.888	0.465	
Neu 10^3^/uL	study	47	1.1	5.49	2.77	2.395	3.19	2.817	0.819	0.708
	control	45	1.21	5	2.61	2.1	3.37	2.759	0.87	
NLR	study	47	0.414	2.729	1.127	0.824	1.423	1.184	0.496	0.002
	control	45	0.644	3.323	1.439	1.057	1.702	1.54	0.623	
PLR	study	47	40.592	208.411	93.171	77.517	116.098	101.087	36.949	<0.001
	control	45	71.512	304.839	131.073	104.886	160	137.991	43.333	
MPV fL *	study	47	9.2	12.5	10.5	10	11.2	10.621	0.855	0.747
	control	45	8.7	13.3	10.6	10.1	11.2	10.684	1.008	
RDW %	study	47	11.3	15.3	12.5	12	13.1	12.636	0.829	0.148
	control	45	11.4	18.5	12.6	12.3	13.3	12.962	1.227	
BMI kg/m^2^ *	study	47	17.31	24.9	21	18.67	22.35	20.79	2.08	0.009
	control	45	18.5	24.8	21.94	20.58	23.12	21.844	1.705	

Abbreviations: SD—standard deviation, CRP—C-reactive protein, Il-6—Interleukin 6, TNF alpha—tumor necrosis factor alpha, AMH—anti-Müllerian, DHEA-S—dehydroepiandrosterone sulfate, FSH—follicle-stimulating hormone, LH—luteinizing hormone, T—testosterone, Cort—cortisol, SHBG—sex-hormone binding globulin, PRL—prolactin, TSH—thyroid-stimulating hormone; WBC—white blood cell, PLT—platelet count, Limf—limphocyte, Neu—neutrophils, NLR—neutrophils/lymphocytes ratio, PLR—platelet/lymphocyte ratio; MPV—mean platelet volume, RDW—red blood cell distribution width; BMI—body mass index; * Student’s *t*-test; the Wilcoxon rank-sum test was used for all other variables.

**Table 3:** Univariate quantile regression models at the 50th percentile assessing the impact of PCOS/PMOS diagnosis on studied variables.

Variables	Estimate	*p*-Value
CRP mg/L	2.864	<0.001
IL-6 pg/mL	1.87	<0.001
TNF alpha pg/mL	1.4	<0.001
AMH ng/mL	3.43	0.001
PRL ng/mL	−7.21	0.001
PLR	−37.903	0.006

Abbreviations: CRP—C-reactive protein, Il-6—Interleukin 6, TNF alpha—tumor necrosis factor alpha, AMH—anti-Müllerian, PRL—prolactin, PLR—platelet/lymphocyte ratio.

**Table 4:** Univariable quantile regression models at the 10th, 50th, and 90th percentiles assessing analyzed variables on AMH concentrations.

Variables	Percentile	Estimate	*p*-Value
CRP mg/L	0.1	0.77	0.001
	0.5	1.16	0.001
	0.9	2.858	<0.001
DHEA-S ug/dL	0.1	0.004	0.275
	0.5	−0.003	0.604
	0.9	−0.018	0.016
FSH IU/L	0.1	−0.057	0.796
	0.5	−0.337	0.29
	0.9	−1.505	0.003
IL-6 pg/mL	0.1	0.947	0.015
	0.5	1.549	0.005
	0.9	2.541	<0.001
LH IU/L	0.1	0.157	0.278
	0.5	0.514	0.02
	0.9	0.985	<0.001
Limf 10^3^/uL	0.1	0.935	0.241
	0.5	0.952	0.396
	0.9	2.848	<0.001
MPV fL	0.1	0.02	0.964
	0.5	0.24	0.688
	0.9	−2.6	0.02
NLR	0.1	0.057	0.938
	0.5	−0.733	0.446
	0.9	−2.307	0.038
Neu 10^3^/uL	0.1	0.158	0.722
	0.5	−0.625	0.323
	0.9	−2.754	0.024
PLR	0.1	−0.007	0.513
	0.5	−0.01	0.436
	0.9	−0.037	0.003
PRL ng/mL	0.1	−0.054	0.367
	0.5	−0.158	0.06
	0.9	−0.362	0.005
RDW %	0.1	−0.026	0.938
	0.5	−0.167	0.73
	0.9	−1.304	0.011
SHBG nmol/L	0.1	0.017	0.312
	0.5	−0.006	0.787
	0.9	−0.068	0.02
TNF alpha pg/mL	0.1	1.594	0.001
	0.5	2.044	0.004
	0.9	4.495	<0.001
glucose 0′ mg/dL	0.1	0.011	0.892
	0.5	−0.145	0.207
	0.9	−0.365	0.013
insulin uU/mL	0.1	−0.027	0.856
	0.5	0.102	0.591
	0.9	0.24	0.028
testosterone ng/mL	0.1	3.858	0.202
	0.5	11.221	0.017
	0.9	25.758	<0.001

Abbreviations: CRP—C-reactive protein, Il-6—Interleukin 6, TNF alpha—tumor necrosis factor alpha, AMH—anti-Müllerian, DHEA-S—dehydroepiandrosterone sulfate, FSH—follicle-stimulating hormone, LH—luteinizing hormone, SHBG—sex-hormone binding globulin, PRL—prolactin, Limf—lymphocyte, Neu—Neutrophils, NLR—neutrophils/lymphocytes ratio, PLR—platelet/lymphocyte ratio; MPV—mean platelet volume, RDW—red blood cell distribution width.

**Table 5:** Multivariable quantile regression models at the 10th, 50th, and 90th percentiles assessing the effects of the investigated variables on AMH concentrations.

Variable	Percentile	Estimate	SE	*p*	95% CI	
TNF alpha pg/mL	0.1	0.220	0.888	0.805	−1.546	1.985
NLR	0.1	0.248	0.786	0.754	−1.315	1.810
PLR	0.1	0.003	0.011	0.772	−0.019	0.026
MPV fL	0.1	0.086	0.442	0.846	−0.793	0.965
Control group	0.1	−1.845	1.287	0.155	−4.404	0.713
TNF alpha pg/mL	0.5	0.166	1.133	0.884	−2.086	2.419
NLR	0.5	0.255	1.165	0.827	−2.060	2.571
PLR	0.5	0.005	0.017	0.760	−0.028	0.038
MPV fL	0.5	0.130	0.615	0.833	−1.093	1.352
Control group	0.5	−3.663	1.889	0.056	−7.418	0.091
TNF alpha pg/mL	0.9	−0.140	1.965	0.944	−4.047	3.767
NLR	0.9	−0.563	1.488	0.706	−3.521	2.395
PLR	0.9	−0.002	0.022	0.920	−0.046	0.042
MPV fL	0.9	−0.171	0.790	0.829	−1.742	1.400
Control group	0.9	−9.927	3.698	0.009	−17.278	−2.576

Abbreviations: SE—standard error; TNF alpha—tumor necrosis factor alpha, NLR—neutrophils/lymphocytes ratio, PLR—platelet/lymphocyte ratio; MPV—mean platelet volume.

## Data Availability

The data presented in this study are available on request from the corresponding author due to privacy and ethical restrictions related to the protection of participants’ personal and clinical data.

## Supplementary Materials

The following supporting information can be downloaded at: https://www.mdpi.com/article/10.3390/biomedicines14102215/s1, Table S1: Multivariable quantile regression models at the 10th, 50th, and 90th percentiles assessing analyzed variables on AMH concentrations. Table S2: Multivariable quantile regression models at the 10th, 50th, and 90th percentiles assessing the effects of the investigated variables on AMH concentrations separately in the study and control groups.
