# Supplementary material for: Is There a Correlation Between Inflammatory Markers and Anti-Müllerian Hormone Level in Non-Obese Patients with Polyendocrine Metabolic Ovarian Syndrome?
Source: Biomedicines. 2026 Sep 30;14(10):2215. doi: 10.3390/biomedicines14102215 (PMC13701418; doi:10.3390/biomedicines14102215)
Supplement: Supplementary file 1 [file biomedicines-14-02215-s001.zip › biomedicines-4535522-supplementary.pdf]

Supplementary table S1. Multivariable quantile regression models at the 10th, 50th, and 90th percentiles assessing analyzed variables on AMH concentrations

Variables	percentile	estimate	p-value
TNF alpha pg/ml	0,1	1,571	**0,002**
NLR	0,1	0,200	0,805
PLR	0,1	0,006	0,574
MPV fL	0,1	0,083	0,854
TNF alpha pg/ml	0,5	2,075	**0,007**
NLR	0,5	0,208	0,871
PLR	0,5	-0,003	0,868
MPV fL	0,5	-0,170	0,794
TNF alpha pg/ml	0,9	3,805	**0,000**
NLR	0,9	2,464	0,102
PLR	0,9	-0,035	0,065
MPV fL	0,9	-0,790	0,254

Legend: TNF-alpha- tumor necrosis factor alpha, NLR- neutrophils/lymphocytes ratio, PLR- platelet/lymphocyte ratio; MPV- mean platelet volume

Supplemantary table S2. Multivariable quantile regression models at the 10th, 50th, and 90th percentiles assessing the effects of the investigated variables on AMH concentrations separately in the study and control groups

Group	variable	percentile	estimate	SE	p	95%CI	
study	TNFalpha pg/ml	0,1	0,275	1,950	0,888	-3,660	4,211
	NLR	0,1	-0,248	2,963	0,934	-6,227	5,731
	PLR	0,1	-0,010	0,042	0,814	-0,094	0,074
	MPV fL	0,1	-0,183	1,350	0,893	-2,908	2,542
	TNFalpha pg/ml	0,5	1,416	2,514	0,576	-3,658	6,490
	NLR	0,5	-0,148	3,935	0,970	-8,089	7,793
	PLR	0,5	-0,001	0,055	0,986	-0,111	0,109
	MPV fL	0,5	-0,524	1,853	0,779	-4,263	3,216
	TNFalpha pg/ml	0,9	3,462	2,787	0,221	-2,162	9,087
	NLR	0,9	2,888	4,263	0,502	-5,715	11,490

	PLR	0,9	-0,050	0,071	0,481	-0,193	0,093
	MPV fL	0,9	-5,188	2,435	**0,039**	-10,102	-0,274
control	TNFalpha pg/ml	0,1	0,229	1,286	0,860	-2,370	2,827
	NLR	0,1	0,468	0,957	0,627	-1,466	2,402
	PLR	0,1	0,001	0,015	0,952	-0,029	0,031
	MPV fL	0,1	0,260	0,549	0,639	-0,850	1,369
	TNFalpha pg/ml	0,5	-1,065	1,451	0,467	-3,997	1,867
	NLR	0,5	0,651	1,171	0,582	-1,716	3,018
	PLR	0,5	-0,002	0,017	0,892	-0,036	0,032
	MPV fL	0,5	0,404	0,574	0,486	-0,757	1,565
	TNFalpha pg/ml	0,9	-2,678	1,545	0,091	-5,800	0,444
	NLR	0,9	0,967	1,300	0,461	-1,661	3,595
	PLR	0,9	-0,019	0,018	0,286	-0,055	0,017
	MPV fL	0,9	0,125	0,601	0,836	-1,090	1,340

Abbreviations: SE-standard error; TNF-alpha- tumor necrosis factor alpha, NLR- neutrophils/lymphocytes ratio, PLR- platelet/lymphocyte ratio; MPV- mean platelet volume;
